# Lichen planus onset during interleukin 23 blockade for treatment of psoriasis

**DOI:** 10.1016/j.jdcr.2025.06.057

**Published:** 2025-08-05

**Authors:** Alia Abbas, Andrew Schroeder, Carly Elston, Chander Raman, Boni Elewski

**Affiliations:** a Department of Dermatology, University of Alabama at Birmingham, Birmingham, Alabama; b UAB Heersink School of Medicine, Birmingham, Alabama

**Keywords:** cytokines, drug reactions, interleukins, lichen planus, psoriasis

## Introduction

Proinflammatory cytokines produced mainly by effector cluster of differentiation 4-positive (CD4^+^) T helper 17 (Th17) cells contribute to the pathogenesis of psoriasis.^1^ Monoclonal antibodies targeting Th17-related cytokines through blockade of interleukin (IL) 17 and IL-23 have become mainstays of psoriasis therapy. Pathogenesis of Lichen Planus (LP) is less understood; however, studies implicate the signal transducer and activator of transcription 1 (STAT1) pathway with IFN-γ and IL-21 noted as the predominant inflammatory cytokines.^2^

Immunotherapy targeting specific cytokines has become standard practice for the treatment of inflammatory dermatologic disease. In some cases, suppression of 1 inflammatory cytokine or pathway leads to the development of a different inflammatory condition that may be mediated by a different CD4^+^ T helper subset/cytokine signature. Herein, we present a patient with recalcitrant psoriasis where treatment with guselkumab (anti–IL-23) could have led to the development of LP that may reflect a shift and/or expansion of STAT1 pathway effector cytokines.

## Case

**Fig 1:**
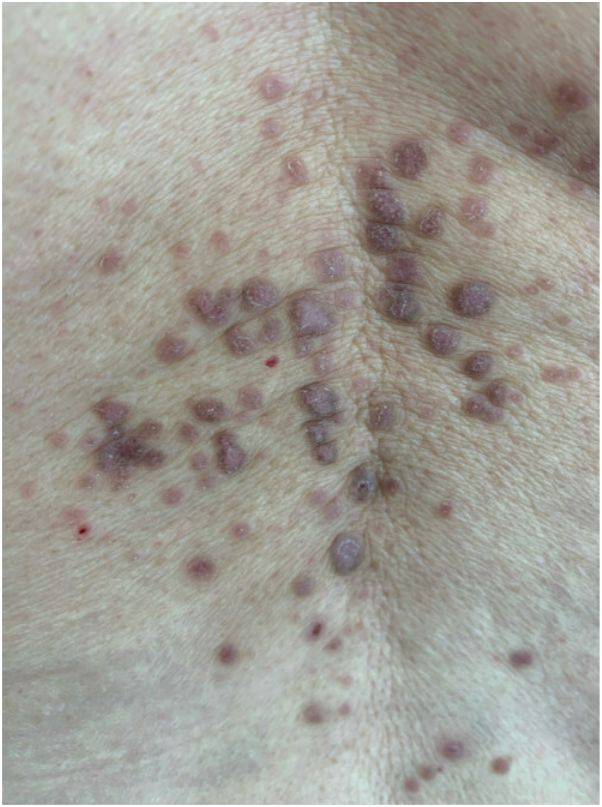
Lichen planus: flat-topped purple polygynal papules with Wickham’s striae on patient’s lower back

**Fig 2:**
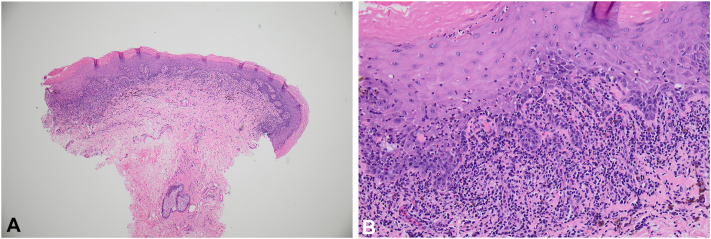
Biopsy of the labia stained with H&E showing compact orthokeratosis overyling an epidermis with irregular sawtooth-like acanthosis and hypergranulosis, robust interface activity with dyskeratosis and civatte bodies, and a band-like lymphocytic infiltrate in the superficial epidermis with pigment incontinence consistent with LP. (**A** and **B,** Hematoxylin-eosin stain; original magnifications: **A,** ×2; **B,** ×20.) *H&E*, Hematoxylin-eosin; *LP*, Lichen Planus.

A 76-year-old woman presented to the clinic with a 1-month history of a new rash while on guselkumab. She had a long-standing history of psoriatic arthritis and psoriasis, which typically occurred on her abdomen, lower back, and extremities. She previously failed many other treatments including methotrexate, adalimumab, ixekizumab, and etanercept but had nearly clear skin on guselkumab for 5 years. The new rash consisted of flat-topped pruritic purple papules with Wickham’s striae on the wrists, lower back, and lower extremities (Fig 1). She also had an erosive white plaque involving the labia minora, perineum, and perianal skin. A punch biopsy from the labia revealed compact orthokeratosis of the epidermis with irregular sawtooth-like acanthosis, hypergranulosis, and lichenoid interface dermatitis consistent with LP. The patient tested negative for both hepatitis B and C. A careful medication review revealed she had been taking valsartan/hydrochlorothiazide for 25 years and meloxicam for 10 years. These medications were discontinued. No other possible drug culprits were identified. Months later, she had another biopsy of the lower back that was also consistent with LP (Fig 2, *A* and *B*). At that time, guselkumab was discontinued. At a subsequent visit, the LP had worsened, but the patient remained in remission from her psoriasis with sustained improvement in her arthritis. The LP continued to worsen, so the patient was started on upadacitinib, which resulted in rapid improvement in her pruritus and LP lesions.

## Discussion

It is well established that Th17 cells are a major pathologic CD4^+^ T helper subset in psoriasis.^1^ The differentiation of Th17 cells is induced by IL-1 β, IL-6, and transforming growth factor-β, although maintenance of Th17 cell populations is dependent on IL-23.^3^ The pathogenesis of LP, on the other hand, is less understood. Evidence points to involvement of IFN-γ and IL-21, which activate STAT1 and Th17 cells, but IL-17 is minimally expressed in LP tissues compared to psoriasis.^2^ Additionally, IL-17 blockade has not been effective in the treatment of LP, arguing against a pathologic role of Th17 cells in this disease.^4^ Our patient developed LP after blockade of IL-23 with guselkumab. In theory, pathologic Th17 cells could have been deprived of IL-23, which would allow a highly active STAT1 to drive the pathogenesis of LP. It is interesting that the development of LP in this patient did not occur until several years into treatment with guselkumab. Although not quite as delayed as this case, a timeframe of up to 2.5 years has been reported in patients with dupilumab-associated psoriasis and psoriasiform manifestations, which are hypothesized to develop due to a similar mechanism.^5^ The possibility of lichenoid drug eruption was also considered but is not favored given worsening of the rash when stopping the suspect drugs and the length of time she had been on both meloxicam (10 years) and valsartan or hydrochlorothiazide (25 years). The average latency for onset of lichenoid drug eruption is 15.7 weeks, with the maximum being 4 years in a review of 323 cases.^6^ Additionally, the idea that the LP could have occurred by chance alone was considered.

Although our conclusion regarding the mechanism is theoretical and other mechanisms of developing LP were explored, our theory is plausible based on the similar phenomenon of psoriasis patients developing atopic dermatitis during IL-17/23 blockade and atopic dermatitis patients developing psoriasis during treatment with dupilumab which blocks IL-4R/13.^5^^,^^7^ More research should be done regarding targeting the JAK/STAT pathway as a therapeutic target for LP and into further elucidating the pathogenesis of inflammation in this disease.

## Conflicts of Interest

Dr Elston is a consultant for and received honorarium from Boerhinger Ingelheim. Dr Elewski receives funding for clinical research through UAB from AbbVie, Anaptys-Bio, Boehringer Ingelheim, Bristol Myers Squibb (BMS), Incyte. Leo, Lilly, Novartis, Pfizer, UCB, and Janssen, and is a consultant for and received honorarium from Amgen, Arcutis, Boehringer Ingelheim, BMS, Leo, Novartis, Pfizer, UCB, Ortho dermatology, and Janssen. Drs Abbas and Schroeder have no conflicts of interest to disclose.
